# Analysis of side population cells derived from dental pulp tissue

**DOI:** 10.1111/j.1365-2591.2010.01789.x

**Published:** 2010-12

**Authors:** M Kenmotsu, K Matsuzaka, E Kokubu, T Azuma, T Inoue

**Affiliations:** 1 Oral Health Science Center HRC7, Tokyo Dental CollageChiba; 2 Department of Clinical Pathophysiology, Tokyo Dental CollegeChiba; 3 Department of Microbiology, Tokyo Dental CollageChiba; 4 Department of Biochemistry, Tokyo Dental CollageChiba, Japan

**Keywords:** ageing, dental pulp, quantitative RT-PCR, side population cells

## Abstract

**Kenmotsu M, Matsuzaka K, Kokubu E, Azuma T, Inoue T.** Analysis of side population cells derived from dental pulp tissue. *International Endodontic Journal*.

**Aim:**

To investigate the characteristics of side population (SP) cells derived from the dental pulp of young and aged rats.

**Methodology:**

Maxillary and mandibular incisors were extracted from 5-week-old (young) rats and 60- to 80-week-old (aged) rats. Coronal pulp tissue was removed mechanically, and single-cell suspensions were prepared using collagenase and dispase. Cells were stained with Hoechst 33342 and sorted with an fluorescence-activated cell sorter (FACS). Isolated SP and main population (MP) cells were analysed by real-time reverse transcription polymerase chain reaction, immunohistochemical localization and cell cycle determination. Two-way analysis of variance and the multiple comparison Scheffè test were used for statistical analysis (*P* < 0.05).

**Results:**

Approximately 0.40% of pulp cells in young rats and 0.11% in aged rats comprised SP cells. SP cells expressed a higher mRNA level of ATP-binding cassette transporter G2 (ABCG2), but lower mRNA levels of nestin, alkaline phosphatase, p16 and p57 than MP cells in both age groups. Immunohistochemical observation revealed ABCG2-positive cells localized in the cell-rich zone and nestin in the odontoblastic layer in both groups. Furthermore, the majority of both young and aged SP and MP cells were in growth arrest of the G_0_/G_1_ phase.

**Conclusion:**

The FACS analysis revealed a decrease in the proportion of SP cells with age, whilst p16 mRNA expression indicated an increase in cell senescence. The cell cycles of SP and MP cells from both young and aged dental pulp were generally in the G0/G1 phase.

## Introduction

Tissue stem (TS) cells have the ability to replicate to maintain the original tissue and can be derived from all types of mature tissues and organs, including dental tissues (Gronthos *et al.* 2000, 2002, Seo *et al.* 2004). TS cells derived from mouse bone marrow are well established and express specific surface markers (Osawa *et al.* 1996). However, TS cells have proven difficult to isolate from other tissues because of the scarcity of specific surface markers. Goodell *et al.* (1996) described a method of distinguishing TS cells using a fluorescence-activated cell sorter (FACS), in which cells were stained with Hoechst 33342, a DNA-binding dye, and sorted at wavelengths of 450 nm and 675 nm by ultraviolet (UV) excitation. This Hoechst 33342-stained cell population was termed Side Population (SP) cells, as it protruded laterally in comparison with the other population, known as main population (MP) cells. SP cells with the ability to efflux Hoechst 33342 have been reported to possess stem cell-like properties. SP cells cannot, however, be detected in the presence of Verapamil as they excrete the dye through ATP-binding cassette (ABC) transporter G2 (ABCG2), which is not expressed by the MP cells (Zhou *et al.* 2001), although several recent studies have reported that ABCG2 is not required for the SP phenotype (Naylor *et al.* 2005, Alt *et al.* 2009). SP cells have been detected in a variety of tissues across species (Goodell *et al.* 1997), including human, bovine, canine and porcine dental pulp (Iohara *et al.* 2006, 2008, 2009). These pluripotent cells have been shown to act as TS cells (Gussoni *et al.* 1999, Iohara *et al.* 2006, 2008, Honda *et al.* 2007). It has been suggested that dental pulp SP cells could differentiate into and be a source of odontoblasts. Moreover, the majority of stem cells could be SP cells, as the stem cell marker ABCG2 is expressed in SP cells (Iohara *et al.* 2006, Honda *et al.* 2007). However, further evidence is required to clarify the findings of these studies.

A reduction in the number and function of somatic cells means that all body tissues undergo change with age (Sherr & DePinho 2000). Previous *in vivo* and *in vitro* studies revealed that dental pulp cells differentiate and are capable of either osteogenesis or dentinogenesis (Inoue *et al.* 1992, Gronthos *et al.* 2000). Reduction in dentinogenic activity and fibrosis progression, loss of cellularity, dystrophic calcification and degeneration of odontoblasts have been reported with age (Bernick & Nedelman 1975, Morse 1991, Nanci 2008). On the other hand, age-related changes in hematopoietic stem cells (HSCs) and bone marrow SP cells have been characterized by an increase in cell number and a reduction in function with age (Beausejour & Campisi 2006, Pearce *et al.* 2007). However, few studies have investigated age-related changes in stem cells and SP cells in dental pulp tissue.

The purpose of this study was to investigate the characteristics of SP cells derived from the dental pulp of young and aged rats.

## Materials and methods

### Animals

All animal studies were conducted in compliance with the Guidelines for the Treatment of Experimental Animals at Tokyo Dental College (Approval Number: 192006). Pulp tissues were obtained from 5-week-old male Sprague Dawley (SD) rats (*n*= 210) weighing 100–120 g each as the young samples and from 60- to 80-week-old male SD rats (*n*= 130) weighing 700–900 g each as the aged samples (Sankyo Lab Service, Tokyo, Japan).

### Sample preparation for FACS and reverse transcription-polymerase chain reaction (RT-PCR) analyses

A large number of pulp cells were necessary for FACS analysis. A total of 20 rats (80 incisors) and 15 aged rats (60 incisors) were sacrificed by cervical dislocation, and both the maxillary and mandibular incisors were extracted and sectioned at the apex to allow removal of the dental germ tissues. Throughout the lifespan of rodents, incisors continuously erupt at the apical end of the tooth, which comprises dental germ tissue. Therefore, in this study, only coronal pulp tissue from the incisor was used, thus excluding dental germ tissue. Each tooth was sectioned in half, and dental pulp tissue was removed mechanically. Removed pulp tissue was minced with scissors and incubated in a solution of 3 mg mL^−1^ collagenase type I (Gibco, Carlsbad, CA, USA) and 4 mg mL^−1^ dispase (Gibco) for 1 h at 37 °C to prepare single-cell suspensions for FACS and RT-PCR analysis.

### Staining of cells with Hoechst 33342 and FACS analysis

Cells were passed through a 40-μm nylon mesh (Cell Strainer; BD Biosciences, San Jose, CA, USA) and suspended at 1.0 × 10^6^ cells mL^−1^ in Hank’s Balanced Salt Solution (HBSS; Sigma-Aldrich, St. Louis, MO, USA) with 2% foetal calf serum (Sigma-Aldrich), HEPES buffer (Gibco) and 1% penicillin/streptomycin (Gibco). Cell suspensions were incubated in a staining medium containing 5 μg mL^−1^ Hoechst 33342 (Sigma-Aldrich) at 37 °C for 90 min in the presence or absence of 50 μmol L^−1^ verapamil (Sigma-Aldrich) and 50 μmol L^−1^ tryprostatin A (Alexis Biochemicals, Carlsbad, CA, USA); verapamil and tryprostatin A are inhibitors of the ABC transporter family. Propidium iodide (Sigma-Aldrich) was added at a concentration of 2 μg mL^−1^ to exclude dead cells. Analysis and cell sorting were performed using an FACS (Aria™; Becton-Dickinson, San Jose, CA, USA). Hoechst 33342 was excited at 350 nm using a UV laser, and fluorescence was measured with 450/50-nm (Hoechst blue) and 530/30-nm band pass (Hoechst red) optical filters. A 505-nm long-pass diachronic mirror was used to separate the emission wavelength. An inhibitor of the ABC transporter family involved in the dye efflux of Hoechst 33342 was used to set the gate for isolation of SP cells. NonSP cells were considered to be MP cells.

### Real-time quantitative RT-PCR analysis

Total RNA was obtained from 1.0 × 10^5^ SP and MP cells using RNeasy Plus Micro Kit (Qiagen, Germantown, MD, USA) according to the manufacturer’s protocol. The quantity of isolated RNA was 8–12 ng mL^−1^ for an RT-PCR run as measured by spectrophotometry (Nano drop® ND-1000; Thermo Fisher Scientific, Wilmington, DE, USA). Total RNA was reverse-transcribed to complementary DNA (cDNA) using the QuantiTect Reverse Transcription Kit (Qiagen). RT-PCR products were analysed by quantitative real-time RT-PCR using TaqMan Gene Expression Assay (Applied Biosystems, Foster City, CA, USA) for the target genes: ABCG2, nestin, alkaline phosphatase (ALP), p16^Ink4a^, p57^Kip2^ and β-actin (internal control), as shown in Table 1. ABCG2 is a stem cell marker, whilst nestin is a neural stem cell and odontoblast cell marker. ALP is an osteoblast differentiation marker. p16^Ink4a^ and p57^Kip2^ are cyclin-dependent kinase inhibitors, which regulate the cell cycle.

**Table 1:** Rat primer for real-time reverse transcription-polymerase chain reaction

Gene	Assay ID	Product size
ABCG2	Rn00710585_m1	94 bp
Nestin	Rn00564394_m1	78 bp
ALP	Rn01516028_m1	68 bp
p16^Ink4a^	Rn00580664_m1	78 bp
p57^Kip2^	Rn00711097_m1	70 bp
β-actin	Rn01768120_m1	63 bp

ABCG2, ATP-binding cassette transporter G2; ALP, Alkaline phosphatase.

PCR was performed using the 7500 fast Real-time PCR System (Applied Biosystems). Gene expression was quantified using TaqMan Gene Expression Assay as the second step in a two-step RT-PCR. Assays were performed in 20 μL single-plex reactions containing TaqMan Fast Universal PCR Master Mix, TaqMan Gene Expression Assays, distilled water and cDNA according to the manufacturer’s protocol (Applied Biosystems). Reaction conditions consisted of a primary denaturation at 95 °C for 20 s and then cycling for 40 cycles of 95 °C for 3 s and 62 °C for 30 s. Expression of selected genes was determined by quantitative RT-PCR in SP and MP cells isolated from young and aged rats. Relative mRNA expression was determined after normalizing cycle threshold values from each gene with the internal control (β-actin) and then using the *Δ*CT values from young MP cells as a reference.

### Sample preparation for cell cycle analysis

Pulp tissues were obtained from young (*n*= 60) and aged (*n*= 45) rats. Experiments were performed in triplicate, using 20 young (80 incisors) and 15 aged (60 incisors) per experiment. Cell preparation and cell sorting were performed in the same manner as for RT-PCR analysis.

### Cell cycle analysis

Side Population and MP cells isolated from dental pulp tissue were pelleted by centrifugation and resuspended in a solution containing 4 mmol L^−1^ sodium citrate (pH 7.6), 0.2% Nonidet P-40 (Roche Diagnosis GmbH, Mannheim, Germany) and 50 μg mL^−1^ propidium iodide (Sigma-Aldrich). After incubation on ice for 30 min, cell suspensions were treated with 0.25 mg mL^−1^ RNase A (Sigma-Aldrich) for 15 min at 37 °C to remove double-stranded RNA. Cells were then analysed by the FACS at an excitation wavelength of 488 nm. Cell cycle analysis was performed in triplicate for both rat groups.

### Optical microscopy

Five young and five aged rats were sacrificed by cervical dislocation, and both the maxillary and mandibular incisors were extracted. Ten extracted mandibular incisors from each group were fixed with 10% neutral buffered formalin and then decalcified with 10% formic acid for 1 week at room temperature. After dehydration with ethanol and embedding in paraffin, a total of 50 paraffin sections of 3-μm were prepared for each group and stained with haematoxylin and eosin. Specimens were observed by light microscopy (Axiophoto 2; Carl Zeiss, Oberkochen, Germany).

**Figure 1:**
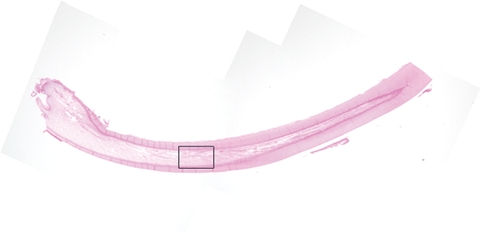
Haematoxylin and eosin staining of sagital section of rat mandibular incisor. Original magnification ×12.5. The area enclosed by the rectangle was further examined by light microscopy.

On light microscopic observation, the rectangle in Fig. 1 was investigated with the area of old- and young-odontoblast classified according to the criteria of Takuma & Nagai (1971) and Murakami *et al.* (2001).

### Immunofluorescence microscopy

Young and aged rats (*n*= 5 each) were sacrificed by cervical dislocation, and their mandibular incisors (10 from each group) were extracted. Dental pulp tissue was removed from the mandibular incisors in the same manner as in the *in vitro* study and immediately frozen in liquid nitrogen. Coronal pulp was removed, washed with phosphate-buffered saline (PBS; Gibco) and incubated with 3% bovine serum albumin (BSA; Sigma-Aldrich) for 30 min at room temperature to prevent nonspecific binding. After removal of BSA, sections were incubated with an anti-ABCG2 rabbit polyclonal antibody (Santa Cruz Biotechnology, Santa Cruz, CA, USA) and an anti-nestin mouse monoclonal antibody (Santa Cruz Biotechnology) overnight at 4 °C. After washing in PBS, the sections were then incubated with the following secondary antibodies for 1 h at room temperature: Alexa Fluor 488 goat anti-rabbit immunoglobulin G (IgG) and Alexa Fluor 555 goat anti-mouse IgG (Molecular Probes, Eugene, OR, USA). The tongue of the same animals, which contains a nonspecific isotype for nestin, was used for the negative control. Specimens treated with only 3% BSA were also used as a control. Specimens were examined and photographed using a confocal laser scanning microscope (LSM 510, Carl Zeiss).

### Statistical analysis

One-way analysis of variance (anova) and the multiple-comparison Scheffè test (*P* < 0.05) were used to compare the proportion of SP cells, the cell cycle phase and mRNA expression in SP and MP cells between the two age groups.

## Results

### Proportion of SP cells in rat dental pulp and cell cycle analysis

**Figure 2:**
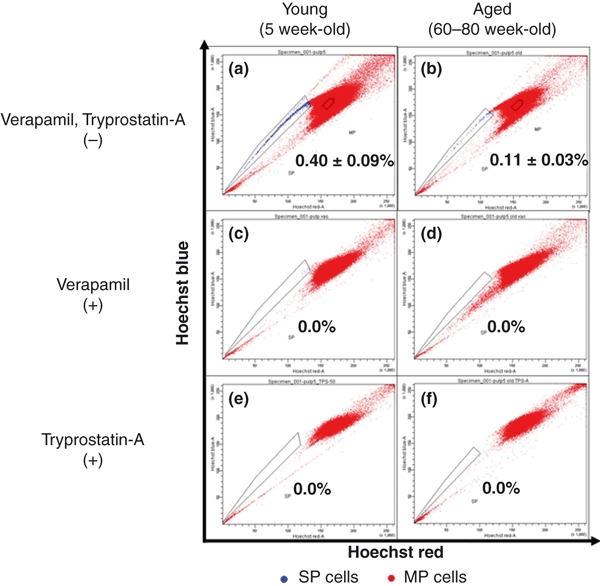
Fluorescence-activated cell sorter (FACS) analysis. Rat pulp cells were analysed for Hoechst 33342 efflux by FACS. From young and aged dental pulp, 0.40% and 0.11% of Side Population (SP) cells were isolated; the proportion was significantly different between groups (*P* < 0.01). The proportion of SP cells decreased with age. The SP cell fraction disappeared with the addition of verapamil and tryprostatin-A.

Young (Fig. 2a) and aged (Fig. 2b) dental pulp was composed of approximately 0.40% and 0.11% SP cells respectively; the proportion of these cells was significantly different between the two age groups (*P* < 0.01). The SP cell fraction disappeared following treatment with verapamil or tryprostatin-A from both young and aged samples (Fig. 2c–f).

**Figure 3:**
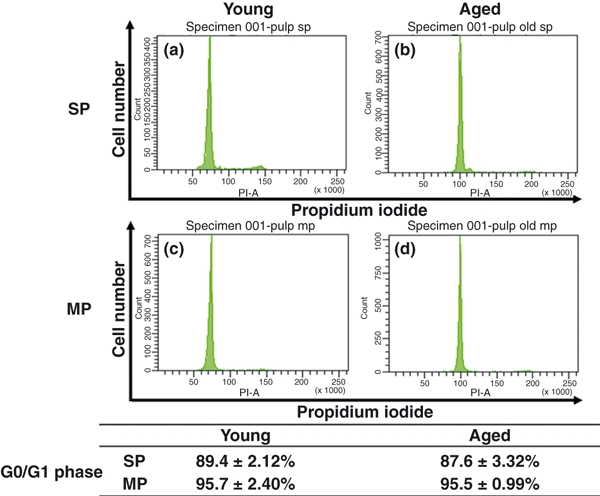
Cell cycle analysis. Most side population (SP) and main population (MP) cells were seen in the G0/G1 phase for both young and aged tissues. Compared with SP cells, young and aged MP cells had a higher percentage in the G0/G1 phase.

The majority of SP and MP cells from both young and aged tissues were in the G0/G1 phase (Fig. 3a–d). A higher proportion of MP cells (95.7 ± 2.40%) and aged (95.5 ± 0.99%) pulp tissues were in the G0/G1 phase compared with SP cells (89.4 ± 2.12% and 87.6 ± 3.32% respectively); the difference between the age groups was not significant.

### Quantitative RT-PCR

**Figure 4:**
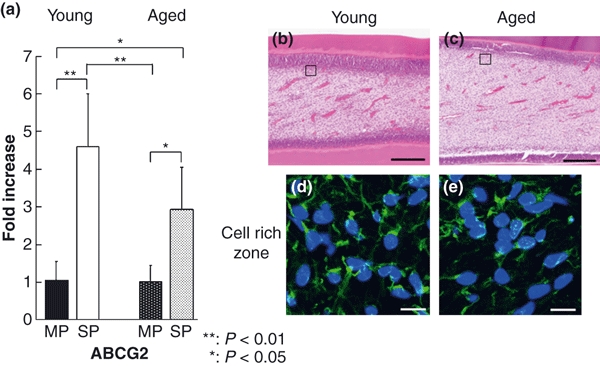
mRNA expression, haematoxylin-eosin staining and immunofluorescence localization of ATP-binding cassette transporter G2 (ABCG2). The expression of ABCG2 mRNA was higher in side population (SP) cells than in main population cells from young and aged pulps (*P* < 0.01, *P* < 0.05) (a). A slightly lower expression of ABCG2 mRNA was observed in aged SP cells; there was no significant difference. Haematoxylin-eosin staining of the area enclosed by the rectangle in Fig. 1 (b and c). Immunofluorescence image of the area enclosed by the square in (b and c). ABCG2-positive cells were observed in the cell-rich zone of young and aged pulp (d and e). Immunofluorescence of ABCG2 (green) was mainly localized on the cell membrane and intercellular structure. Scale bar: 200 μm (b and c), 10 μm (d and e).

ABCG2 mRNA expression was higher in SP than in MP cells of both young and aged pulps (*P* < 0.01, *P* < 0.05) (Fig. 4a). A slight decrease in ABCG2 mRNA expression was observed in aged SP cells compared with that in young SP cells, although the difference was not significant. ABCG2 mRNA expression in MP cells of both young and aged pulps was not significantly different. Moreover, young SP cells showed higher expression than aged MP cells, and aged SP cells showed higher expressions than young MP cells (*P* < 0.01, *P* < 0.05).

**Figure 5:**
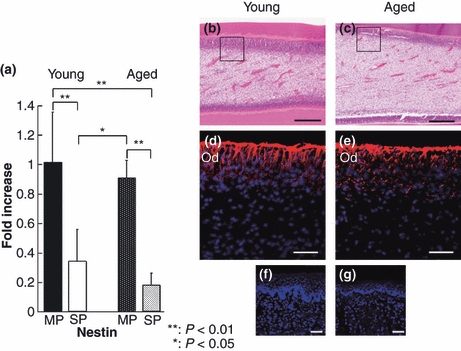
mRNA expression, haematoxylin-eosin staining and immunofluorescence localization of nestin. The expression of nestin mRNA was significantly lower in side population (SP) cells than in main population (MP) cells from both young and aged pulp (*P* < 0.01) (a). A slightly lower expression of nestin was observed in aged SP and MP cells; however, there was no significant difference. Haematoxylin-eosin staining of the area enclosed by a rectangle in Fig. 1(b and c). Immunofluorescence image of the area enclosed by the square in (b and c). Immunofluorescence of nestin (red) is expressed at the odontoblast layer in young and aged dental pulp (b and c). Intense immunoreactivity for nestin tended to be found in aged dental pulp, compared to young dental pulp. As a negative control, tongue of the same animals was examined (f and g). OD: odontoblast layer, scale bar: 200 μm (b and c), 50 μm (d, e, f and g).

Expression of nestin mRNA was lower in SP than in MP cells from both young and aged pulp (*P* < 0.01) (Fig. 5a). A slight decrease in expression of nestin mRNA was observed in both aged SP and MP cells in comparison with young SP and MP cells, although the difference was not significant. Moreover, the expression of nestin mRNA was lower in young SP cells than in aged MP cells and lower in aged SP cells than in young MP cells (*P* < 0.05, *P* < 0.01).

**Figure 6:**
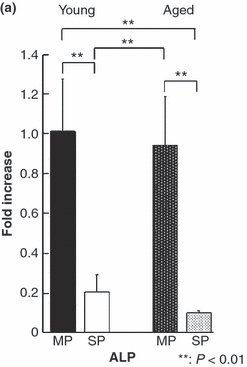
Alkaline phosphatase (ALP) mRNA expression. The expression of ALP mRNA was significantly lower in side population cells than in MP cells from young and aged tissue (*P* < 0.01). There was no significant difference between age groups.

Expression of ALP mRNAs was lower in SP than in MP cells from both young age groups (*P* < 0.01) (Fig. 6). No significant differences were observed between young and aged cells in either cell type. Expression of ALP mRNA was lower in young SP cells than in aged MP cells and lower in aged SP cells than in young MP cells (*P* < 0.01).

**Figure 7:**
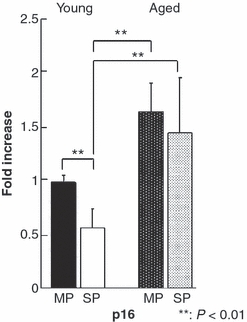
p16 mRNA expression. The expression of p16 mRNA was lower in young side population (SP) cells than in young main population (MP) cells (*P* < 0.01). Aged cells expressed a higher level of p16 mRNA than young SP and MP cells. The expression of p16 was higher in aged SP cells than in young SP cells (*P* < 0.01).

Expression of p16 mRNA was significantly lower in young SP than in MP cells (*P* < 0.05) but not significantly different between aged SP and MP cells (Fig. 7). The aged cells expressed a higher level of p16 mRNA than the young cells independent of the type of cell. A slight decrease in p16 mRNA expression was also observed in young SP and MP cells compared to their aged counterparts, although the difference was not significant. Moreover, the p16 mRNA expression was higher in aged SP cells than in young SP cells (*P* < 0.05) and young MP cells (*P* < 0.01), but lower in young SP cells than in aged MP cells (*P* < 0.01).

**Figure 8:**
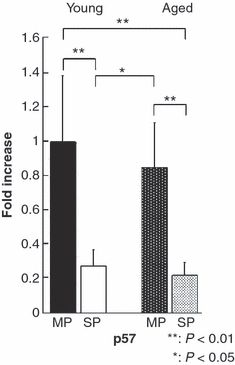
p57 mRNA expression. The expression of p57 mRNA was lower in side population cells than in main population cells, from both young and aged tissue (*P* < 0.01). There was no significant difference between age groups.

Expression of p57 mRNA was lower in SP than in MP cells from both age groups (*P* < 0.01) (Fig. 8); no significant difference was observed between the groups. A slight decrease in expression of p57 mRNA was observed in both aged SP and MP cells when compared with young SP and MP cells, although the difference was not significant. Expression of p57 mRNA was lower in young SP cells than in aged MP cells (*P* < 0.05) and lower in aged SP cells than in young MP cells (*P* < 0.01).

### Histological observations

On histological observation, blood vessels, nerves and cells such as odontoblasts and fibroblasts comprised the rat dental pulp tissue, showing no significant difference between the young and aged groups (Fig. 4b, c).

### Immunohistochemical observations

ABCG2-positive cells were located in the cell-rich zone of both young and aged dental pulp (Fig. 4d, e). ABCG2 was mainly localized on the cell membrane.

Nestin-positive cells were concentrated in the odontoblastic layer in both young and aged groups (Fig. 5d, e).

## Discussion

### Characterization of SP cells

As SP cells from mice bone marrow were first established by Goodell *et al.* (1996), SP cells have been reported in many body organs (Zhang *et al.* 2005, Challen *et al.* 2006, Uezumi *et al.* 2006, Umemoto *et al.* 2006, Yamahara *et al.* 2008). However, the SP cell proportion varies depending on the species, tissue and organ. Although this difference in proportion is particularly marked in oral tissues (Iohara *et al.* 2006, Kawanabe *et al.* 2006, Honda *et al.* 2007), the SP cell proportion has been reported to be 0.2% in porcine dental pulp (Iohara *et al.* 2006) and 0.8% in cultured human dental pulp (Honda *et al.* 2007). In this study, young rat dental pulp SP cells had an approximately 0.40% proportion of SP cells, which is similar to the proportions or less than the 1% observed in several other organs; however, the proportion of SP cells in organs varies greatly. This discrepancy may be a tissue-specific phenomenon. Moreover, this study found a significant difference (*P* < 0.01) in the proportion of SP cells between young and old rats, approximately 0.40% and 0.11%, respectively. Nanci (2008) reported that undifferentiated mesenchymal stem cells revealed a decrease in number with age, as seen here whilst Garvin *et al.* (2007) reported a decrease in SP cell numbers in human bone marrow with age. On the other hand, Pearce *et al.* (2007) found that numbers of HSCs and SP cells increased with age in mice and concluded that if the functionality of stem cells decreased with age, then the increased number of cells and maintained quality would compensate for the absence of exclusive function.

### Relationship between SP cells and ABCG2

ABCG2 is a member of the ABC transporter super family and was first described in drug-resistant MCF-7/Adr cells (Doyle *et al.* 1998). It is a half-transporter and is responsible for the Hoechst dye efflux pattern in the SP cell fraction, which is enriched with stem and/or progenitor cells in various tissues (Zhou *et al.* 2001, Bunting 2002, Scharenberg *et al.* 2002, Pfister *et al.* 2008). The SP cell fraction disappeared in cells treated with ABC transporter family inhibitors, verapamil and tryprostatin-A. Verapamil is a multi-drug resistance gene inhibitor, whilst tryprostatin-A is an ABCG2-specific inhibitor, which does not inhibit another important ABC transporter, P-glycoprotein (Woehlecke *et al.* 2003). SP cell analysis of limbal epithelial cells revealed that tryprostatin-A effectively inhibits Hoechst 33342 dye efflux from those cells (Watanabe *et al.* 2004). In this study, quantitative RT-PCR analysis showed high levels of ABCG2 mRNA expression in SP cells from both young and aged rat pulp tissues. Immunohistochemical observation also showed ABCG2-positive cells mainly observed in the cell-rich zone both young and aged dental pulp. In support of these findings, a low level of ABCG2 mRNA expression was also detected in both young and aged MP cells. On the other hand, many reports suggest that the ABCG2 transporter is neither required nor responsible for the SP phenotype in many type of cells from various species (Naylor *et al.* 2005, Morita *et al.* 2006, Alt *et al.* 2009). Further study is therefore necessary to further elucidate the relationship between ABCG2 and SP cells.

### Relationship between SP cells and nestin

Nestin is a marker for neural stem cells (Lendahl *et al.* 1990) and has previously been detected in SP cells isolated from tissues such as retina (Bhattacharya *et al.* 2003), limbal epithelium (Umemoto *et al.* 2006), odontoblast and tooth germs (Terling *et al.* 1995, About *et al.* 2000). Nestin may, therefore, be a characteristic cytoskeletal molecule not only in neural stem cells, but also in other adult stem cells with the SP cell phenotype. Recently, Honda *et al.* (2007) have reported a higher expression level of nestin mRNA in SP cells than in MP cells derived from cultured human dental pulp. In contrast, this study found higher nestin mRNA expression in MP cells than in SP cells derived from both young and aged pulp. As nestin was immunohistochemically positive in odontoblasts, MP cells most likely contain large numbers of odontoblasts, thereby explaining the higher expression of nestin in MP cells than in SP cells.

### Differentiation of SP cells

ALP is an enzyme expressed in the early stage of mineralization and is a marker of pulp cell viability (Inoue *et al.* 1992). It is known to be localized in odontoblasts and in the cells of the cell-rich zone. In this study, MP cells expressed higher levels of ALP mRNA than SP cells from both young and aged tissues. These observations suggest that even though pulp SP cells have the capacity for dentinogenesis, they may be at an earlier stage of differentiation than MP cells.

### Cell cycle and ageing of SP cells

A defining feature of HSCs is that they reside in the G0/G1 phase in the cell cycle, which represents the quiescent state (Uchida *et al.* 1997, Arai *et al.* 2004). Bone marrow and limbal epithelium SP cells are also found in the G0/G1 phase (Umemoto *et al.* 2005, 2006). Maintenance of SP cells in the G0/G1 phase is controlled by p57 (Umemoto *et al.* 2005, Yamazaki *et al.* 2006). In this study, both young and aged SP and MP cells were found mainly in the G0/G1 phase, suggesting slowed proliferation or growth arrest. Casasco *et al.* (1997) reported that about 90% of human dental pulp tissue is in the G0/G1 phase. This study found similar results with the proportion of total young and aged SP and MP cells in the G0/G1 phase calculated to be approximately 90%; there was no significant difference between the two types of cells.

Expression of p57 mRNA in MP cells was much higher than that in SP cells from young and aged tissues. These results suggest that MP cells obtained from the pulp are stable independent of age, most likely because this tissue is enclosed in the pulp chamber where metabolism is reduced (Muramatsu *et al.* 2005, Nanci 2008).

The phenomenon whereby cell division is stopped permanently after a certain number of cell divisions is called cell senescence (Hayflick & Moorhead 1961). During cell senescence, the telomeres are shortened after each cell division. Cell senescence in rodents involves not only telomere shortening, but also CDK inhibition through inhibitors like p16 (Kiyono *et al.* 1998). These inhibitors bind directly to CDK or the cyclin-CDK complex to suppress the progress of the cell cycle. Identified as a molecule that binds to CDK4, p16 inhibits the catalytic activity of CDK4/cyclin D enzymes (Serrano *et al.* 1993). Expression of p16 increases as the cell ages, reducing the capacity for self-replication (Beausejour & Campisi 2006, Kim & Sharpless 2006). Taken together, SP cells are most likely altered with ageing. On the other hand, expression of ABCG2, nestin and ALP showed no significant differences between the young and aged groups in this study, although nestin and ALP expression slightly decreased with age in both SP and MP cells.

In this study, the mRNA expression level of these factors showed no significant difference between young and aged tissue samples, although the protein expression level remains to be examined.

## Conclusions

Fluorescence-activated cell sorter analysis revealed a decrease in the proportion of SP cells in rat dental pulp tissue with age. Expression of p16 mRNA, an ageing marker, increased in SP cells from dental pulp tissue with age. Young and aged SP and MP cells were generally found in the G0/G1 phase. No significant differences were observed in the characteristics of SP cells between young and aged dental pulp, apart from gene expression of p16.
